# A retrospective analysis of 20,178 adult neurological infection admissions to United Kingdom critical care units from 2001 to 2020

**DOI:** 10.1186/s12879-024-08976-z

**Published:** 2024-01-25

**Authors:** Joseph Donovan, Abena Glover, John Gregson, Andrew W. Hitchings, Emma C. Wall, Robert S. Heyderman

**Affiliations:** 1 Clinical Research Department, London School of Hygiene and Tropical Medicine, Keppel St, London, UK; 2 University College London Hospitals NHS Trust, London, UK; 3 St George’s University Hospitals NHS Trust, London, UK; 4 St George’s, University of London, London, UK; 5 The Francis Crick Institute, London, UK; 6 University College London Hospitals NIHR Biomedical Research Centre, London, UK; 7 Research Department of Infection, Division of Infection and Immunity, University College London, London, UK

**Keywords:** Neurological infection, Meningitis, Encephalitis, Critical care, Adults

## Abstract

**Background:**

Neurological infection is an important cause of critical illness, yet little is known on the epidemiology of neurological infections requiring critical care.

**Methods:**

We analysed data on all adults with proven or probable neurological infection admitted to UK (NHS) critical care units between 2001 and 2020 reported to the Intensive Care National Audit and Research Centre. Diagnoses, physiological variables, organ support and clinical outcomes were analysed over the whole period, and for consecutive 5-year intervals within it. Predictors of in-hospital mortality were identified using a backward stepwise regression model.

**Results:**

We identified 20,178 critical care admissions for neurological infection. Encephalitis was the most frequent presentation to critical care, comprising 6725 (33.3%) of 20,178 cases. Meningitis– bacterial, viral or unspecified cases - accounted for 10,056 (49.8%) of cases. In-hospital mortality was high, at 3945/19,765 (20.0%) overall. Over the four consecutive 5-year periods, there were trends towards higher Glasgow Coma Scale scores on admission, longer critical care admissions (from median 4 [IQR 2–8] to 5 days [IQR 2–10]), and reduced in-hospital mortality (from 24.9 to 18.1%). We identified 12 independent predictors of in-hospital death which when used together showed good discrimination between patients who die and those who survive (AUC = 0.79).

**Conclusions:**

Admissions with neurological infection to UK critical care services are increasing and the mortality, although improving, remains high. To further improve outcomes from severe neurological infection, novel approaches to the evaluation of risk stratification, monitoring and management strategies are required.

**Supplementary Information:**

The online version contains supplementary material available at 10.1186/s12879-024-08976-z.

## Key points

• Meningitis comprised 50% and encephalitis comprised 33% of neurological infections requiring critical care admission.

• During the 20-year study period, there was a progressive trend of increasing neurological infection admissions to critical care, and a reduction in the overall mortality rate.

## Introduction

Neurological infections are major causes of mortality and morbidity [1]. Worldwide, meningitis and encephalitis are estimated to cause 290,000 and 108,000 deaths per year, respectively [1, 2]. The mortality rates of neurological infections, which can exceed 50% in some settings, are higher than those of most other infection types [2–4]. Neurological infections frequently present as emergencies, which may rapidly progress to death, or permanent neurological disability in survivors [3, 5, 6]. In the UK, patients with neurological infection are managed in both specialist and non-specialist hospitals. Uncertainties in risk stratification, diagnostic approaches, monitoring strategies, and optimal management particularly of severe disease and raised intracranial pressure (ICP), likely contribute to lengthy hospital admissions and a substantial healthcare burden.

Patients with neurological infection often require admission to critical care units for close monitoring and physiological support, aiming to minimise secondary brain injury [6, 7]. Support includes close neurological monitoring, seizure prophylaxis, and airway protection in those with depressed consciousness. Less commonly, neurological infection may develop in individuals already in critical care, particularly those who have undergone invasive neurological procedures [8].

There are limited data to guide management of neurological infections beyond use of anti-infectives and corticosteroids [7, 9–13]. Raised ICP is often suspected in severe disease, yet its incidence in primary neurological infection is uncertain given invasive ICP monitoring is not routine. Improved outcomes with ICP-guided management have been described in some settings [14, 15]. In an observational study of 52 adults with bacterial meningitis in Sweden, mortality at 2 months was significantly lower in the group receiving ICP-guided therapy (target ICP < 20 mmHg and cerebral perfusion pressure > 50 mmHg), vs. standard care [15]. Cases were not randomly assigned to groups, and the extent to which the study results were related to ICP control, as opposed to other inter-group differences, is uncertain. Non-invasive ICP monitoring may make such an approach more widely deliverable.

Neurological infections account for a minority of critical care admissions, but are associated with high mortality. In the 24-hour point prevalence study EPIC III, conducted at 1150 centres in 88 countries in 2017, of 8135 patients in intensive care with suspected or proven infection, 314 (3.9%) had neurological infection [16]. Their in-hospital mortality was 29.0%. Few studies have captured the full spectrum of neurological infection in critical care [17, 18]. In a prospective multicenter international cohort study of meningoencephalitis in intensive care, of 591 cases with identified aetiologies there were 247/591 (41.8%) cases of acute bacterial meningitis and 140/591 (23.7%) cases of infectious encephalitis [19]. The characteristics, management and outcomes of neurological infection in UK critical care have not been well characterized.

A National Infection Trainees Collaborative for Audit and Research study of community acquired meningitis in the UK and Ireland estimated that 50% patients with confirmed bacterial meningitis required critical care admission [20]. To address this knowledge gap, and characterize adult neurological infection admissions to UK critical care, we analyzed records from the Intensive Care National Audit and Research Centre (ICNARC) [21], an independent charity that collects and provides national-level data of UK critical care admissions. In this retrospective cohort study, we investigated the burden, type, presentation, severity, organ support requirements, and clinical outcomes, of adult neurological infection admissions to UK critical care units over a 20-year period.

## Methods

### Case selection

Data were retrieved for adults (≥ 18 years) admitted to UK critical care units, with reasons for critical care admission (primary, secondary, and ultimate reasons using the ICNARC Coding Method, in the ICNARC Case Mix Programme [CMP] database) coded as encephalitis, meningococcal meningitis, meningitis unspecified, bacterial meningitis not meningococcal, intracranial abscess, infected cerebrospinal fluid (CSF) shunt, viral meningitis, and tuberculous meningitis. Cerebral malaria cases could not be separated from non-cerebral malaria, and were excluded. Cases of spinal infection were not included. One or more diagnoses were provided for each case, assigned either at critical care admission or discharge. Following ICNARC methodology, the primary reason for admission was considered to be the most important underlying condition or reason for admission to the critical care unit, assessed and recorded at admission to (and during the first 24 h in) the critical care unit. For cases where ‘meningitis unspecified’ was recorded with a second neurological infection diagnosis, the more precise diagnosis was used. For cases with more than one neurological infection diagnosis (other than ‘meningitis unspecified’) e.g., ‘encephalitis’ plus ‘viral meningitis’, the diagnosis listed as the ‘primary reason for admission’ was used. If this was not available, the ‘secondary reason for admission’ was taken, or the ‘ultimate reason for admission’ if neither was available.

ICNARC data were collected under existing ethical approvals (Supplementary Material 1). Ethical approval for this study was granted by the London School of Hygiene and Tropical Medicine Research Ethics Committee (26,550).

### Clinical data

Data were identifed based on the ICNARC data collection form 3.1 (2015). The following parameters were obtained: age, gender, weight, height, date of hospital admission, date of critical care admission, source of critical care admission, reason for critical care admission, and comorbidities. Lowest and highest values during the first 24 h in critical care were obtained for: temperature, blood pressure, heart rate, respiratory rate and urine output; hemoglobin concentration, white cell count and platelet count; and serum concentrations of bicarbonate, sodium, potassium, glucose, lactate, urea, and creatinine. Arterial blood gas parameters for lowest pH and lowest PaO_2_ were obtained. Details of pupil reactivity, lowest Glasgow Coma Scale (GCS) score, period of sedation and paralysis, and highest level of physiological support provided, the number of days of organ support (by organ system), dates of critical care unit discharge, and hospital discharge, were also obtained.

‘Days of neurological support’ was defined as the number of calendar days (including part days) during which any neurological support was administered. Neurological support was defined as: a requirement for monitoring for central nervous system depression prejudicing the airway and its protective reflexes; invasive neurological monitoring or treatment; continuous intravenous medication to control seizures; continuous cerebral monitoring; or therapeutic hypothermia.

### Statistical analysis

Data were summarized using medians for continuous variables and counts (with percentages) for discrete and ordinal variables. Requirement for neurological support, disability and mortality outcomes were described for all cases, and then stratified by admission brain infection type, and four consecutive 5-year admission periods (2001–2005, 2006–2010, 2011–2015, 2016–2020) chosen prior to data analysis.

We aimed to identify independent predictors of mortality by building a mortality prediction model. We considered all variables listed in Table 1 for inclusion, but excluded variables for which data were missing in > 1,000 cases (with the exception of GCS which was judged likely to be a very strong predictor of mortality). For variables with multiple measurements within a 24-hour period, we investigated associations with both the lowest and highest values, as defined by the ICNARC risk prediction model [22]. We expected some variables to have non-linear relationships with mortality (e.g., body temperature). To assess the appropriate form with which to include these variables we first assessed univariate associations of all continuous predictors using restricted cubic splines with 3 knots. We identified which variables were significant predictors by including them in a logistic regression model with a backwards stepwise selection procedure, with p values ≥ 0.05 eliminated at each iteration. We report odds ratios (OR) of death during hospital admission, and 95% confidence intervals (CIs). The ability of variables to discriminate between death and survival was assessed using the C-statistic, equivalent to the area under the receiver operating characteristic (ROC) curve. Analysis was performed using STATA version 17 (StataCorp *LLC*, College Station, TX) and R version 4.2.2 (R Foundation for Statistical Computing, Vienna, Austria) (Supplementary Material 2).

**Table 1:** Critical care admission, and clinical/laboratory parameters during the first 24 h

Characteristic	Summary	
	Patients with data	Median (IQR) or N(%)
Age (years)	20,176	53 (37, 66)
Sex	20,178	
- Male		11,086 (54.9%)
- Female		9092 (45.1%)
Body mass index	14,136	25.7 (22.9, 29.4)
Type of admission	20,178	
- Encephalitis		6725 (33.3%)
- Meningitis, unspecified		5360 (26.6%)
- Bacterial meningitis, non-meningococcal		3105 (15.4%)
- Intracranial abscess		2730 (13.5%)
- Meningococcal meningitis		1096 (5.4%)
- Infected CSF shunt		665 (3.3%)
- Viral meningitis		363 (1.8%)
- Tuberculous meningitis		132 (0.7%)
Time from hospital admission to critical care unit admission (days)	20,177	0 (0, 1)
Location prior to critical care unit admission *	16,464	
- Accident and emergency		6961 (42.3%)
- Hospital ward		5225 (31.7%)
- Theatre or recovery		1801 (10.9%)
- Level 3 ICU		1280 (7.8%)
- Recovery as temporary critical care		308 (1.9%)
- HDU equivalent bed		233 (1.4%)
- Level 2 HDU		226 (1.4%)
- Imaging department		199 (1.2%)
- Enhanced care (non-HDU/ICU)		139 (0.8%)
- Obstetrics		35 (0.2%)
- Not in the hospital		31 (0.2%)
- Outpatients		1 (0%)
- Paediatrics		1 (0%)
Past medical history *	19,965	
- Chemotherapy		410 (2.1%)
- Steroid treatment		308 (1.5%)
- Chronic renal replacement therapy		288 (1.4%)
- HIV		279 (1.4%)
- Metastatic disease		172 (0.9%)
- Radiotherapy		158 (0.8%)
- Lymphoma		148 (0.7%)
- Severe respiratory disease		106 (0.5%)
- Biopsy proven cirrhosis		100 (0.5%)
- AML or ALL or multiple myeloma		93 (0.5%)
- Hepatic encephalopathy		86 (0.4%)
- CML or CLL		81 (0.4%)
- Very severe cardiovascular disease		77 (0.4%)
- Congenital immuno-humoral or cellular immune deficiency state		48 (0.2%)
- Home ventilation		20 (0.1%)
Baseline observations ^#^		
- Lowest temperature (^o^C)	20,021	36.1 (35.6, 36.6)
- Highest temperature (^o^C)	19,769	37.7 (37.0, 38.4)
- Lowest blood pressure (mmHg)	20,062	97 (86, 109)
- Highest blood pressure (mmHg)	20,019	155 (140, 174)
- Lowest heart rate (bpm)	20,059	68 (57, 80)
- Highest heart rate (bpm)	20,008	105 (90, 120)
- Lowest non-ventilated respiratory rate (breaths per min)	11,549	14 (12, 18)
- Lowest ventilated respiratory rate (breaths per min)	13,439	12 (12, 15)
- Highest ventilated respiratory rate (breaths per min)	12,116	18 (15, 23)
Arterial blood gas – lowest pO_2_		
- pO_2_ (KPa)	17,734	10.7 (9.2, 12.5)
- FiO_2_	17,721	0.30 (0.25, 0.40)
- PaCO_2_ (KPa)	17,732	4.9 (4.4, 5.6)
- pH (KPa)	17,726	7.41 (7.36, 7.45)
Arterial blood gas – lowest pH		
- PaCO_2_ (KPa)	17,480	5.5 (4.8, 6.3)
- pH	17,479	7.36 (7.30, 7.40)
Baseline blood tests ^#^		
- Lowest serum bicarbonate (mmol/l)	7891	22.0 (19.4, 24.5)
- Highest serum bicarbonate (mmol/l)	6116	24.7 (22.4, 27.0)
- Lowest serum sodium (mmol/l)	19,479	137 (134, 140)
- Highest serum sodium (mmol/l)	15,351	141 (138, 144)
- Lowest serum potassium (mmol/l)	19,421	3.7 (3.3, 4.0)
- Highest serum potassium (mmol/l)	15,405	4.3 (4.0, 4.6)
- Lowest serum creatinine (µmol/l)	19,327	69 (53, 94)
- Highest serum creatinine (µmol/l)	13,770	80 (62, 116)
- Lowest serum glucose (mmol/l)	16,287	6.1 (5.1, 7.4)
- Highest serum glucose (mmol/l)	14,321	9.2 (7.4, 11.6)
- Highest blood lactate (mmol/l)	14,183	1.6 (1.1, 2.6)
- Highest serum urea (mmol/l)	19,039	6.0 (4.2, 9.0)
- Lowest white blood cell count (x10^9^/L)	19,320	11.7 (8.1, 17.0)
- Highest white blood cell count (x10^9^/L)	13,641	15.1 (10.5, 21.3)
- Lowest platelet count (x10^9^/L)	19,071	199 (140, 270)
Urine output (mls)	19,692	1984 (1292, 2920)
Glasgow coma score (/15)	12,035	10 (7, 14)
Pupil reactivity	13,999	
- Left pupil reactive to light		12975 (93.7%)
- Right pupil reactive to light		12952 (93.6%)
Sedated and ventilated	20,108	
- Sedated for all of first 24 hours		7073 (35.2%)
- Paralysed and sedated for all of first 24 hours		451 (2.2%)
- Paralysed and sedated for some of first 24 hours		5865 (35.2%)
- Not sedated and ventilated		6719 (35.2%)

N = number of patients for whom data are available, included in that statistic. Summary statistic = the median (1st and 3rd quartile) value for continuous data, and the number and frequency (%) of patients with the characteristic for categorical data. Age is defined as age of admission to critical care unit, derived from date of birth and date of admission to critical care unit. Body mass index is derived from weight and height data (kg/m^2^). *For location prior to critical care unit admission, and past medical history, individual options are listed as defined as per ICNARC data collection manual 3.1 (2013). ^#^Lowest and highest admission parameters (baseline observations and baseline blood tests), as selected by ICNARC [22], are defined as lowest and highest values during the first 24 h of critical care unit admission, respectively. ‘Temperature’ represents central temperature. ‘Lowest blood pressure’ represents lowest systolic blood pressure with paired diastolic reading. ‘Highest blood pressure’ represents highest systolic blood pressure with paired diastolic reading. ALL = acute lymphocytic leukaemia. AML = acute myeloid leukaemia. bpm = beats per minute. CLL = chronic lymphocytic leukaemia. CSF = cerebrospinal fluid. HDU = high dependency unit. HIV = Human immunodeficieny virus. ICNARC = Intensive Care National Audit and Research Centre. ICU = intensive care unit. Min = minute

## Results

### Critical care admission

Neurological infection represented 20,178 (0.7%) of 2,808,359 adult admissions to all UK critical care units participating in the ICNARC CMP from 1st January 2001 to 31st January 2020. Critical care observation time was for a total of 416.3 years (20,169 cases). The median age at critical care unit admission was 53 years (interquartile range [IQR] 37–66), and 11,086 cases (54.9%) were male. Neurological infection diagnoses requiring critical care admission were: encephalitis, 6725 (33.3%); meningitis unspecified, 5360 (26.6%): bacterial meningitis non-meningococcal, 3105 (15.4%); intracranial abscess, 2730 (13.5%); meningococcal meningitis, 1096 (5.4%); infected CSF shunt, 665 (3.3%); viral meningitis, 363 (1.8%); tuberculous meningitis, 132 (0.7%). Additional admission diagnoses of status epilepticus or uncontrolled seizures, secondary hydrocephalus and tuberculosis are described in Supplementary Material 3. The emergency department was the most common source of admission (6961 [42.3%]). Critical care admission data, including clincial and laboratory parameters evaluated during the first 24 h in critical care, are described in Table 1.

### Organ support

The median duration of critical care unit stay was 4 days (IQR 2–9). The median durations of level 3 support (two or more organ systems, or advanced respiratory support) [23] and level 2 support (single organ) were 3 days (IQR 1–7), and 2 days (IQR (0–3), respectively. 6624/16,449 (40.3%) required at least one day of neurological support. The neurological infections that most commonly required neurological support were CSF shunt infection (398/569 [69.9%] cases), intracerebral abscess (1200/2400 [50.0%] cases), and tuberculous meningitis (65/132 [49.2%] cases). Overall, 325/547 (59.4%) of cases with an CSF shunt infection, 1051/2228 (47.2%) of cases with an intracranial abscess, and 51/116 (44.0%) of cases with tuberculous meningitis, were expected to require at least minor assistance with some daily activities in the two weeks following hospital discharge.

## Outcomes

The median length of hospital stay was 20 days (IQR 10–44). Overall, 3945/19,765 (20.0%) cases with neurological infection died in the hospital in which the critical care unit was based (Table 2). The neurological infection type associated with highest mortality during hospital admission was tuberculous meningitis (44/127 [33.3%]). For those discharged from hospital alive, at least minor assistance was required with daily activities in the first two weeks in 325/547 cases (59.4%) of CSF shunt infection, 1051/2228 cases (47.2%) of intracranial abscess, and 51/116 cases (44.0%) of tuberculous meningitis.

**Table 2:** Outcome and neurological support, for all patients and by brain infection type

Characteristic	All patients		Encephalitis		Meningococcal meningitis		Meningitis, unspecified		Intracranial abscess		Infected CSF shunt		Bacterial meningitis, non-meningococcal		Viral meningitis		Tuberculous meningitis	
			N	Statistic	N	Statistic	N	Statistic	N	Statistic	N	Statistic	N	Statistic	N	Statistic	N	Statistic
Neurological support required	16,449	6624 (40.3%)	5700	2154 (37.8%)	1091	333 (30.5%)	3097	1122 (36.2%)	2400	1200 (50.0%)	569	398 (69.9%)	3095	1234 (39.9%)	363	117 (32.2%)	132	65 (49.2%)
Neurological support days (days)	16,449	0 (0, 2)	5700	0 (0, 2)	1091	0 (0, 1)	3097	0 (0, 2)	2400	0.5 (0, 3)	569	3 (0, 8)	3095	0 (0, 2)	363	0 (0, 1)	132	0 (0, 6)
Mortality during hospital admission	19,765	3945 (20.0%)	6588	1293 (19.2%)	1084	211 (19.3%)	5256	1117 (20.8%)	2665	527 (19.3%)	635	83 (12.5%)	3047	618 (19.9%)	361	51 (14.1%)	127	44 (33.3%)
Expected dependency post discharge*	14,770		5174		920		2750		2228		547		2704		330		116	
- No assistance		8959 (60.7%)		2953 (57.1%)		687 (74.7%)		1801 (65.5%)		1177 (52.8%)		222 (40.6%)		1825 (67.5%)		229 (69.4%)		65 (56.0%)
- Minor assistance required		3431 (23.2%)		1333 (25.8%)		153 (16.6%)		581 (21.1%)		601 (27.0%)		143 (26.1%)		537 (19.9%)		62 (18.8%)		21 (18.1%)
- Considerable assistance required		1595 (10.8%)		591 (11.4%)		57 (6.2%)		235 (8.5%)		329 (14.8%)		132 (24.1%)		204 (7.5%)		28 (8.5%)		18 (15.5%)
- Total assistance required		491 (3.3%)		191 (3.7%)		12 (1.3%)		74 (2.7%)		79 (3.5%)		41 (7.5%)		80 (3.0%)		8 (2.4%)		6 (5.2%)
- Expectation of dying		294 (2.0%)		106 (2.0%)		11 (1.2%)		59 (2.1%)		42 (1.9%)		9 (1.6%)		58 (2.1%)		3 (0.9%)		6 (5.2%)

Neurological support data decribe neurological support for all patients with available data, including those for whom no neurological support was required. Of those individuals requiring at least 1 day of neurological support, a median of 3 days (IQR 2–7) were required. N = number of patients for whom data are available, included in that statistic. Statistic = the median (1st and 3rd quartile) value for continuous data, and the number and frequency (%) of patients with the characteristic for categorical data. *Assessed at critical care unit discharge, this assesses the expected dependency in the two weeks following discharge from the acute hospital. CSF = cerebrospinal fluid

### Analysis by consecutive time periods

**Fig. 1:**
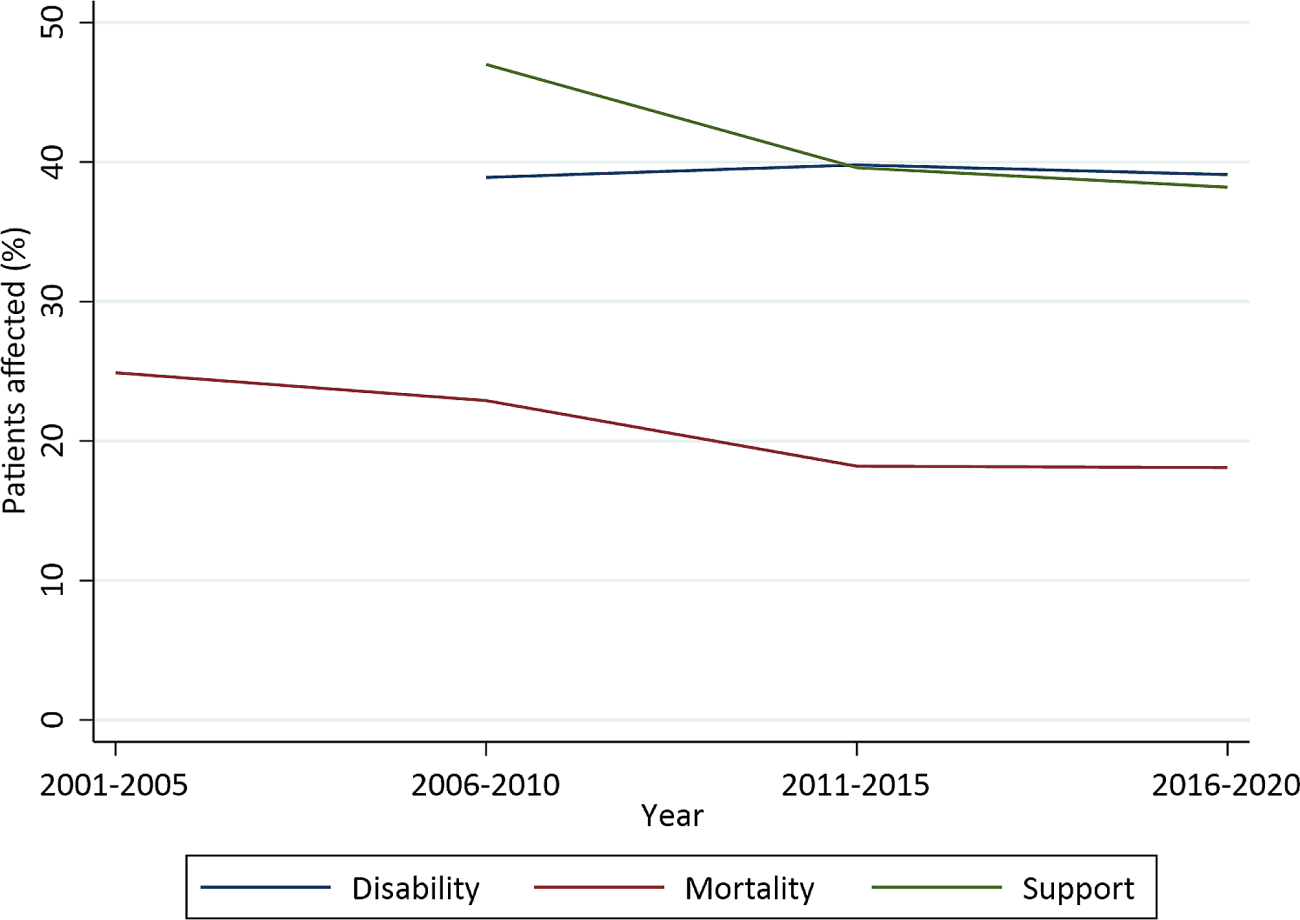
Outcomes and neurological support, by period of admission (5-year periods). N = Total number of patients in dataset for this time period. Neurological support requirement, and expected dependency (disability) data not available for 2001–2005 period

Neurological infection admissions to critical care reported to ICNARC increased from 2742 in 2001–2005 to 7339 in 2015–2020. The number of critical care units reporting data to the ICNARC CMP also increased, from a median of 170 units to 283 units over the same time period. In-hospital mortality was 24.9% in 2001–2005, falling progressively to 18.1% in 2016–2020, and there was a similar reduction in the requirement for neurological support (Table 3; Fig. 1). Lowest GCS, days to critical care admission, and days of critical care stay, are described for the 5-year intervals of this study in Table 3. Over the four consecutive 5-year periods, the proportion of cases dying during the admission fell; the admission GCS increased; and the duration of admissions lengthened (Table 4).

**Table 3:** Outcomes and critical care admission, by period of admission (5-year periods)

Characteristic	2001–2005		2006–2010		2011–2015		2016–2020	
	N	N (%) or median (IQR)	N	N (%) or median (IQR)	N	N (%) or median (IQR)	N	N (%) or median (IQR)
Neurological support required	Data on neurological support unavailable for this time period		2869	1347 (47.0%)	6241	2474 (39.6%)	7339	2803 (38.2%)
Neurological support days (days)			2869	0 (0, 3)	6241	0 (0, 2)	7339	0 (0, 2)
Mortality during hospital admission	2654	662 (24.9%)	3755	861 (22.9%)	6144	1119 (18.2%)	7212	1303 (18.1%)
Expected dependency post discharge *	Data on dependency unavailable for this time period		2536		5628		6606	
- No assistance				1550 (61.1%)		3386 (60.2%)		4023 (60.9%)
- Minor assistance required				579 (22.8%)		1339 (23.8%)		1513 (22.9%)
- Considerable assistance required				273 (10.8%)		620 (11.0%)		702 (10.6%)
- Total assistance required				85 (3.4%)		173 (3.1%)		233 (3.5%)
- Expectation of dying				49 (1.9%)		110 (2.0%)		135 (2.0%)
Age at critical care admission (years)	2742	49 (32–62)	3855	50 (35–64)	6242	54 (38–66)	7339	55 (40–67)
Time between hospital and critical care admission (days)	2741	0 (0–1)	3855	0 (0–1)	6242	0 (0–2)	7339	0 (0–2)
Duration of critical care stay (days)	2735	4 (2–8)	3853	4 (2–8)	6242	4 (2–9)	7339	5 (2–10)
Lowest GCS recorded during first 24-hour period in critical care (/15)	1471	9 (5–14)	2269	10 (6–14)	3830	11 (8–14)	4465	11 (8–14)

N = number of patients for whom data are available, included in that statistic. Statistic = the median (1st and 3rd quartile) value for continuous data, and the number and frequency (%) of patients with the characteristic for categorical data. Time blocks represent January 1st to 31st December inclusive (for example 2016–2020 represents 1st January 2016 to 31st December 2020 inclusive). *Assessed at critical care unit discharge, this assesses the expected dependency in the two weeks following discharge from the acute hospital. GCS = Glasgow coma score

**Table 4:** Diagnosis by period of admission (5-year blocks)

	2001–2005 *N* = 2742	2006–2010 *N* = 3855	2011–2015 *N* = 6242	2016–2020 *N* = 7339
Encephalitis	716 (26.1%)	1101 (28.6%)	2091 (33.5%)	2817 (38.4%)
Meningococcal meningitis	0 (0.0%)	245 (6.4%)	411 (6.6%)	440 (6.0%)
Meningitis, unspecified	1689 (61.6%)	1295 (33.6%)	1235 (19.8%)	1141 (15.5%)
Intracranial abscess	262 (9.6%)	456 (11.8%)	935 (15.0%)	1077 (14.7%)
Infected CSF shunt	75 (2.7%)	120 (3.1)	207 (3.3%)	263 (3.6%)
Bacterial meningitis, not meningococcal	0 (0.0%)	578 (15.0%)	1205 (19.3%)	1322 (18.0%)
Viral meningitis	0 (0.0%)	59 (1.5%)	145 (2.3%)	159 (2.2%)
Tuberculous meningitis	0 (0.0%)	0 (0.0%)	12 (0.2%)	120 (1.6%)

N = number of patients for whom data are available, included in that statistic. The number and frequency (%) of patients with the characteristic are shown. CSF = cerebrospinal fluid

### Mortality prediction

**Fig. 2:**
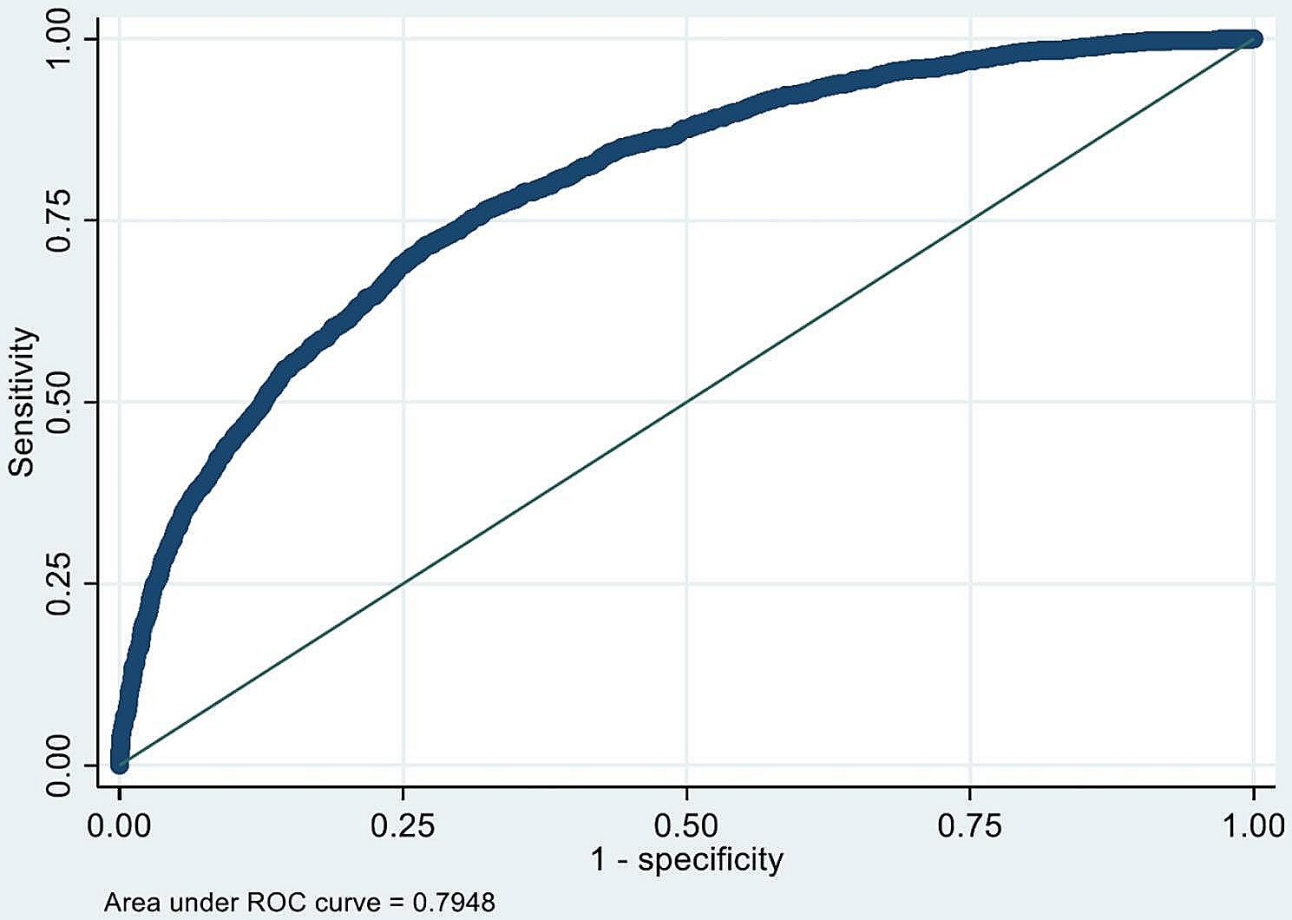
Receiver operating characteristic curve for 12-variable mortality prediction. ROC = receiver operating characteristic

A total of 10,403 admissions had complete information on all variables considered for inclusion (listed in the footnote to Table 5). Among this subset of patients, we identified 12 variables each of which were independently and significantly associated with increased risk of mortality in multivariate models. The odds of death during hospital admission were lower for females (OR vs. males: 0.84, 95% CI 0.75, 0.94, *p* < 0.01), and increased with age (OR 1.2 [CI 1.2, 1.3] per decade older, *p* < 0.001). Three or more co-morbidities (from the list shown in Table 1) strongly predicted death during hospital admission (OR vs. 0 or 1 comorbidities 2.4, 95% CI 1.8, 3.4, *p* < 0.001). For each 1-point increase in the lowest GCS score in the first 24 h of admission, the odds of death reduced by 0.84 (95% CI 0.82, 0.85, *p* < 0.001). Tachycardia (> 90 beats/minute), hypotension (systolic blood pressure < 110mmHg), leukopenia (< 25 × 10^9^/L and tachypnea were each associated with increased mortality. Low serum urea (below 10mmol/l) was associated with reduced risk. For several variables there was a U-shaped association with mortality. For minimum body temperature the lowest risk occurred at 37^o^C, and for maximum body temperature it occurred at 38^o^C. For urine output the lowest risk was at 2.5 L/day, and for serum sodium it was at 140mmol/L. Inclusion of all 12 measurements in a mortality prediction model gave a C-statistic of 0.79 (Fig. 2), indicating that an admission who dies has a higher predicted risk than an admission who survives on 79% of occasions.

**Table 5:** Odds ratios for in-hospital mortality 24 h after critical care admission

Variables	Odds ratio of death during hospital admission	95% confidence interval	P value
Female vs. male	0.84	0.75, 0.94	< 0.01
Age, per decade older	1.23	1.19, 1.28	< 0.001
Co-morbidities			< 0.001
None or 1	1.00	Reference category	
2	1.39	1.14, 1.71	
3 or more	2.44	1.75, 3.40	
Lowest total Glasgow Coma score	0.84	0.82, 0.85	*P* < 0.001
Body temperature			
Minimum temperature, per ^0^C below 37^0^C	1.22	1.13, 1.32	< 0.001
Minimum temperature, per ^0^C above 37^0^C	1.56	1.19, 2.05	< 0.01
Maximum temperature, per ^0^C below 38^0^C	1.24	1.11, 1.39	< 0.001
Maximum heart rate per 10 beats per minute above 90	1.20	1.17, 1.23	< 0.001
Minimum systolic blood pressure per 10mmHg below 110mmHg	1.09	1.05, 1.14	< 0.001
Minimum respiratory rate (10 breaths per min)	1.42	1.23, 1.63	< 0.001
Urine output			
per litre below 2.5 L	1.31	1.20, 1.44	< 0.001
per litre above 2.5 L	1.30	1.22, 1.38	< 0.001
Minimum serum sodium			
per 10mmol/l below 140mmol/l	1.29	1.13, 1.46	< 0.001
per 10mmol/l above 140mmol/l	1.79	1.49, 2.14	< 0.001
Maximum serum urea per 1mmol/l below 10mmol/l	0.93	0.90, 0.95	< 0.001
Minimum white blood cell count per 1 × 10^9^/L below 25 × 10^9^/L	1.03	1.01, 1.05	< 0.05

Analysis includes 10,403 patients with complete information on the following co-variates: age, sex, reason for admission (type of infection), number of comorbidities, temperature, blood pressure, heart rate, minimum respiratory rate (ventilated or unventilated), serum sodium, serum potassium, serum urea, white blood cell count, platelet count, urine output, sedation/ventilation used

## Discussion

Harnessing the high-quality ICNARC database, we present the largest descriptive analysis of neurological infection amongst adults admitted to critical care units in the UK over 20 years. Taken together, encephalitis was the most common diagnosis requiring admission to critical care. Meningitis diagnoses– including bacterial, viral, and unspecified cases - accounted for almost 50% of admissions. Typically, patients were admitted to critical care within 24 h of presentation, most commonly from the emergency department. Critical care stays were short, and almost half of cases required neurological support. Over 20 years there was a trend towards higher admission GCS scores, longer critical care stays, and reduced in-hospital mortality.

Neurological infections such as bacterial meningitis and viral encephalitis in adults are uncommon causes of admission to hospital in the UK, but have disproportionately high morbidity and mortality burden. In our cohort, which is expected to include individuals with the most severe disease, the in-hospital mortality of 20% was similar to the 23.9% mortality rate reported from a broader, multinational observational study of patients admitted to 167 ICUs in 17 European countries [24]. UK data are sparse; a recent study of community acquired meningitis admissions to UK and Ireland hospitals reported 3% in-hospital mortality [20]. However, patients with encephalitis, cryptococcal tuberculous and nosocomial meningitis were excluded from the analysis, and only 192 (13.1%) patients required ICU admission.

An analysis of mortality by sequential 5-year time periods demonstrated a progressive reduction of mortality over time; from 24.9% in 2001–2005, to 18.1% in 2016–2020. While a change in ICU admission criteria cannot be excluded, this is consistent with improved outcomes from neurological infection over the past 25 years [25], and is likely to be associated with significant improvements in diagnostics, monitoring, treatments, and critical care management over this time period. Furthermore, the Global Burden of Disease Study for neurological disorders (195 countries, 1990–2016) demonstrated both mortality, and disability-adjusted life-years, are improving for meningitis and encephalitis [25], The health improvements responsible for these data may also be driving fewer severe neurological infection presentations to critical care.

Our data describe a steady increase in the reporting of neurological infection critical care admissions to the ICNARC CMP. This contrasts with the reduction in disease severity, particularly severe meningococcal disease, which might be anticipated from the introduction of national vaccination programmes which started with MenC in 1999 [26]. The proportion of UK critical care neurological infection admissions diagnosed with meningococcal meningitis has actually remained stable during the period of this analysis. Overall, critical care admissions were longer, which may reflect an increasing need for prolonged critical care support, a more cautious approach to step-down, or improved survival amongst the study population. The observed increase in admissions may be explained by several factors which are not mutually exclusive. These include improved data collection with an increase in the number of critical care units reporting data over this period; lower thresholds for critical care admission of neurological infections, perhaps reflecting an increase in the number of critical care beds available in the UK (from 3.8 to 5.9 per 100,000 people between 2011 and 2020) [27]; improved recognition and diagnosis of neurological infection in the community or in-hospital, through better diagnostics (especially CSF multiplex PCR), improved brain imaging for encephalitis and rapid neurological reporting, and clinical practice guidelines for neurological infection [8, 27, 28], and finally an increase in severe neurological infections in a growing and ageing UK adult population [28]. An increase in admission GCS during the study period may also support lower thresholds for critical care admission of neurological infections.

In our analysis, encephalitis was the most common neurological infection diagnosis, accounting for more cases than meningococcal meningitis and non-meningococcal bacterial meningitis combined. This is consistent with estimates describing a higher incidence of encephalitis than bacterial meningitis in the UK [29, 30]. Our study is large and the first to describe the UK burden of neurological infections requiring critical care. However, due to its observational and retrospective design, it has limitations. We describe a requirement for neurological support in ~ 40% cases, but the nature of support required by individual patients is unknown. The ICNARC coding method did not specifically identify cryptococcal disease as a cause of meningitis, and these presentations may be represented by other meningitis codes in the data. Our analysis assumes all meningitis and encephalitis cases are caused by infection, and might erroneously include some non-infectious causes. Neurological infection diagnoses are dependent upon accurate clinical coding, and may not reflect microbiological data. Misclassification of cases is possible, particularly for distinction between viral meningitis and encephalitis, and between viral and bacterial meningitis. Prior to 2006, specific meningitis codes were not used, and meningitis cases were grouped together. We combine data for neurological infections that differ in pathophysiology and treatment. No disability data were reported for 2001–2005, so the time-period trend data in this respect are limited. Data were reported to ICNARC on a voluntary basis. Increasing participation in the ICNARC CMP over the study period affects evaluation of changing dynamics over this time. Consecutive 5-year intervals were chosen to give four equal-length periods that would contain sufficient cases to draw conclusions; however, a different approach (e.g., 2-year groups) may have revealed different trends.

Notwithstanding its limitations, our analysis provides a comprehensive picture of the burden of neurological infections managed in UK critical care units, including changes in its characteristics and outcomes over a 20-year period. Our analysis shows that mortality remains high. In this context, the gaps in the evidence to inform management strategies are notable, particularly the uncertainties in risk stratification, the optimal use and benefits of intracranial pressure monitoring and case management. These knowledge gaps hamper our ability to select the most appropriate cases for critical care; to allocate them rationally to specialist and non-specialist units; and to provide the care they need to secure the best possible outcomes.

### Electronic supplementary material

Below is the link to the electronic supplementary material.

Supplementary Material 1

## Data Availability

The data that support the findings of this study are available from the Intensive Care National Audit and Research Centre (ICNARC) but restrictions apply to the availability of these data, which were used under license for the current study, and so are not publicly available. Data are however available from the authors upon reasonable request and with permission of ICNARC.
