# Supplementary material for: A retrospective analysis of 20,178 adult neurological infection admissions to United Kingdom critical care units from 2001 to 2020
Source: BMC Infect Dis. 2024 Jan 25;24:132. doi: 10.1186/s12879-024-08976-z (PMC10809450; doi:10.1186/s12879-024-08976-z)
Supplement: Supplementary file 1 — Supplementary Material 1 [file 12879_2024_8976_MOESM1_ESM.docx]

**Supplementary material**

Supplementary material 1

As viewable on the ICNARC website (https://www.icnarc.org/Our-Audit/Audits/Cmp/About/Governanceapprovals), ICNARC have approval to hold patient identifiable data under section 251 of the NHS Act 2006. Approval is granted by the Confidentiality Advisory Group (CAG) within the Health Research Authority (HRA) (previously National Information Governance Board). The approval number for the CMP is PIAG 2-10(f)/2005.

Approvals are available on the HRA website and attached below.

https://www.hra.nhs.uk/planning-and-improving-research/application-summaries/confidentiality-advisory-group-registers/

Select "2001 - 2008 approved applications (advice provided by PIAG)"

Search "PIAG 2-10(f)/2005"

Supplementary Material 2

These analyses were performed with R (version 4.2.2), and with STATA (version 17).

The following R packages were used: binGroup, broom, dplyr, ggbreak, ggplot2, ggpubr, ggrepel, gridExtra, Hmisc, kableExtra, knitr, lubridate, psych, rmarkdown, stringi, tidyr

The following STATA packages were used: dm91, pmcalplot, estout

Supplementary Material 3

Amongst 20,178 admissions to critical care with neurological infection, 1062 (5.3%) had an additional admission reason of ‘status epilepticus or uncontrolled seizures’ (605 [57.0%] encephalitis, 18 [1.7%] meningococcal meningitis, 172 [16.2%] meningitis unspecified, 112 [10.5%] intracerebral abscess, 19 [1.8%] infected cerebrospinal fluid shunt, 95 [8.9%] bacterial meningitis non meningococcal, 37 [3.5%] viral meningitis, and 4 [0.4%] tuberculous meningitis. 393 (1.9%) admissions had an additional admission reason of ‘secondary hydrocephalus’ (31 [7.9%] encephalitis, 14 [3.6%] meningococcal meningitis, 88 [22.4%] meningitis unspecified, 79 [20.1%] intracerebral abscess, 68 [17.3%] infected cerebrospinal fluid shunt, 75 [19.1%] bacterial meningitis non meningococcal, 5 [1.3%] viral meningitis, and 33 [8.4%] tuberculous meningitis. 70 (0.3%) admissions had an additional admission reason of ‘tuberculosis’ (of whom 26 [37.1%] received a diagnosis of meningitis unspecified, 18 [25.7%] a diagnosis of bacterial meningitis non meningococcal, and just 11 [15.7%] tuberculous meningitis.
